# A Global Database of Soil Plant Available Phosphorus

**DOI:** 10.1038/s41597-023-02022-4

**Published:** 2023-03-07

**Authors:** R. W. McDowell, A. Noble, P. Pletnyakov, P. M. Haygarth

**Affiliations:** 1 AgResearch, Lincoln Science Centre, Private Bag 4749, Christchurch, 8140 New Zealand; 2 Faculty of Agriculture and Life Sciences, Lincoln University, Lincoln, P O Box 84, 7647 Christchurch, New Zealand; 3 Lancaster Environment Centre, Lancaster University, Lancaster, LA1 4YQ UK

**Keywords:** Agriculture, Element cycles, Environmental impact

## Abstract

Soil phosphorus drives food production that is needed to feed a growing global population. However, knowledge of plant available phosphorus stocks at a global scale is poor but needed to better match phosphorus fertiliser supply to crop demand. We collated, checked, converted, and filtered a database of *c*. 575,000 soil samples to *c*. 33,000 soil samples of soil Olsen phosphorus concentrations. These data represent the most up-to-date repository of freely available data for plant available phosphorus at a global scale. We used these data to derive a model (*R*^*2*^ = 0.54) of topsoil Olsen phosphorus concentrations that when combined with data on bulk density predicted the distribution and global stock of soil Olsen phosphorus. We expect that these data can be used to not only show where plant available P should be boosted, but also where it can be drawn down to make more efficient use of fertiliser phosphorus and to minimise likely phosphorus loss and degradation of water quality.

## Background & Summary

Soil phosphorus drives food production required to feed an increasing global population that is projected to reach 10 billion people by 2050^1^. It has been estimated that an additional 500 million hectares of arable land will be required to feed this increased population unless phosphorus can be either better utilised by plants or applied more efficiently^2^. Much of this efficiency will arise from local management solutions that only apply phosphorus fertilisers where they are needed^3^. However, knowledge of plant-available soil phosphorus stocks is poor, globally.

Some estimates have been made of global soil total phosphorus but only considers soils in their natural state, that is without the addition of fertilisers^4,5^. Similarly, regional estimates exist of plant available soil phosphorus stocks using measured data^6–8^. However, global estimates of plant available soil phosphorus stocks using measured data do not exist. Instead, global stocks have been estimated using models of factors such as plant uptake, weathering and global lithology data^9–12^ or via mass balance approaches^2,13^. It is important to know where available soil phosphorus concentrations are adequate or deficient for optimal crop growth. This knowledge enables us to better match phosphorus fertiliser supply to crop demand and to suggest where excess plant available soil phosphorus can be drawn down^11,14^. Here we present the first global database of freely available data on plant available soil phosphorus concentrations and use these data to create a global map and calculate the global stocks of plant available soil phosphorus stocks. We chose bicarbonate-extractable Olsen phosphorus^15^ as the measure of plant available soil phosphorus as it is the most widely used form, globally.

## Methods

### Data filtering and evaluation

**Fig. 1:**
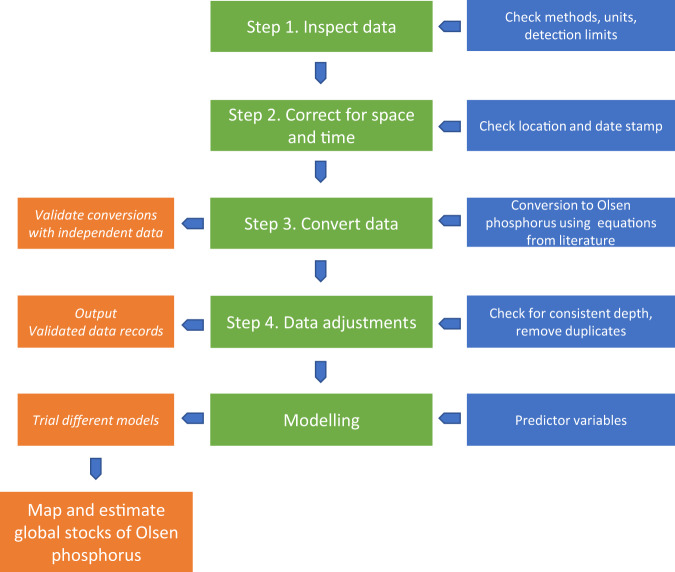
Flowchart of the steps involved in filtering, evaluation, and modelling of soil Olsen phosphorus data. Note that the blue and orange boxes are sub tasks associated with each step and resulting outputs, respectively.

Data (n = 574,375) of available soil phosphorus were obtained from 19 regional or global databases and published studies. These were chosen for their geographic spread and representativeness of a mix of developed and developing nations and where there was a clear process in place to ensure that data were of good quality (Table 1). Prior to modelling the data to estimate global Olsen phosphorus stocks, we adopted a multi-step process (Fig. 1) to produce a globally consistent dataset. The steps comprised (1) inspecting the data and filtering it for consistent analytical methods, units, and a limit of detection (set as 2 mg kg^−1^); (2) filtering data to remove points lacking correct geo-referencing and those falling outside an acceptable time span (from 2000–2019); (3) converting values into Olsen phosphorus concentrations via established equations (Table 2), if necessary; and 4) filtering data to remove points from depths >20 cm and eliminating any duplicate values.

**Table 1:** List of data sources used to construct the map of the estimated global soil Olsen phosphorus (P) stock.

Data source (short name)	Number of samples^a^	Year^b^	Conversion of filtered and evaluated data into Olsen phosphorus data^c^	Coverage	References
International Soil Reference and Information Centre (ISRIC) World Soil Information	42,026 (2,776)	2010 (2010)	2,461 converted from Bray-I P, 129 converted from Mehlich-3 P	Global	^40^
Global dataset of plant-available P (Hou)	215 (176)	1982–2018 (1992)	176 converted from Resin-P	Global	^5^
Land Use/Land Cover Area Frame Survey (LUCAS) Topsoil Survey	19,969 (15,472)	2009 (2009)		Europe	^41^
National Cooperative Soil Survey (NCSS)	427,238^d^ (1,759)	1956–2018 (2002)	1,105 converted from Bray-I P, 370 converted from Mehlich-3 P	US and global	^42^
Soil database for land surface modelling (CTSDB)	1,096 (518)^e^	1980–1996 (1996)		China	^18,43^
Chinese Ecosystem Research Network (CERN)	8,106 (125)	1998–2010 (2009)		China	^44^
World Soil Information Service (WoSIS)	56,162 (6,822)	2009–2016 (2011)	4,515 converted from Bray-I P, 1,043 converted from Mehlich-3 P	Africa	^6^
Soil Resources of Russia (SRR)	4,961 (145)	2010–2019 (2014)	145 converted from Kirsanov^f^	Russia	^45^
Australian Soil Resources Information System (ASRIS)	15,525 (4,789)	2011 (2011)	4,209 converted from Bray-I P, 12 converted from Mehlich-3 P	Australia	^46^
New Zealand Soil database (NZSD)	243 (106)	1995–2009 (2004)		New Zealand	^47^
Miscellaneous (Misc)	253 (253)	1990–2017 (2011)	65 converted from Bray-I P, 50 converted from AB-DPTA	Bangladesh, Myanmar, Nepal, Pakistan, West Africa	^16,48–55^
All	575,794 (32,941)	1982–2019 (2009)	19,695 required no conversion	—	—

^a^The numbers in the parentheses refer to the filtered number of data.

^b^The values in the parentheses refer to the average sampling year of the filtered data.

^c^No conversion was necessary if blank; otherwise, please refer to Table 2 for the conversion details.

^d^Original data were pre-filtered to the top 15 cm soil layer (the 2012 data points are then further filtered).

^e^Data sourced from eastern provinces (Anhui, Fujian, Guangdong, Guangxi, Hainan, Hebei, Jiangsu, Jiangxi, Shandong, Sichuan, and Tianjin) and prior to 1995 are removed.

^f^No publications exist to convert Kirsanov available P^56^ into Olsen phosphorus^57^. We employ the weighted mean equation for Bray-I as the closest approximation of the 0.2 N HCl extractant used in the Kirsanov test.

**Table 2:** Regression equations to convert Mehlich-3, Bray-I and Resin phosphorus into Olsen phosphorus for calcareous and non-calcareous soils.

Soil test/pH range	Soil characteristics	Number of samples	Jurisdiction	Slope	Intercept	R^2^	Reference
**Conversion from Mehlich-3**^**a**^							
4.3–6.8	Non-calcareous	91	US	0.33	6.9	0.88	^58^
5.3–8.2	Non- calcareous	~180	US	0.47	1.2	0.79	^59^
	Calcareous	~60	US	0.45	1.8	0.81	^59^
3.8–8.6	Non-calcareous	66	Italy	0.70	−0.6	0.71	^60^
	Calcareous	54	Italy	0.37	0.4	0.73	^60^
**Weighted mean**							
	Non-calcareous	337		0.47	2.4	0.80	
	Calcareous	114		0.41	1.1	0.77	
Conversion from Bray-I							
5.3–7.45	Non-calcareous	~180	US	0.43	2.4	0.68	^59^
6.0–8.0	Non-calcareous	165	US	0.68	3.9	0.95	^61^
4.3–6.8	Non-calcareous	91	US	0.30	2.9	0.85	^58^
**Weighted mean**		436		0.49	3.1	0.82	
Conversion from AB-DPTA							
6.7–9.1	^b^	35	India	1.81	4.1	0.50	^62^
**Conversion from Resin**							
5.1–7.7	Non-calcareous	59	US	0.71	0.1	0.91	^63^
	Calcareous	30	US			0.94	^64^

To convert the data into Olsen phosphorus data, we use slope and intercept values calculated as weighted mean values for the calcareous and non-calcareous FAO soil types^25^.

^a^We consider the conversion equation of Zbíral and Němec^65^ to be too geographically limited (Czech Republic only) and too variable to be included.

^b^Unknown. Soil characteristics were not listed, and no site-specific coordinates were available to independently check them.

#### Step 1 Inspect data

When examining data, we determined that the soil extraction method was recorded, and that the phosphorus extraction relied on acceptable procedures. Measurements of phosphorus based on molybdenum blue colorimetry or ion chromatography were considered comparable and acceptable. Measurements obtained with the stannous chloride method were excluded from the database. We also inspected the data for irregularities such as different units or different detection limits. The units were restricted to mg kg^−1^, and volumetric data (mg L^−1^) were excluded. Where detection limits were reported (2 mg kg^−1^), minimum values were expressed as half the detection limit (1 mg kg^−1^). Where detection limits were not reported, we inspected each data source for repeated low concentrations and assigned detection limits equivalent to half of the values that were repeatedly reported. Values at or below the detection limit comprised < 0.1% of the final database.

#### Step 2 Correct for space and time

We determined whether data points were correctly geo-referenced and occurred within an acceptable time span. To increase the likelihood that data points were correctly geo-referenced we excluded any data that were incorrectly reported or located in aquatic systems, glaciers, or permanent snowpacks. To generate an acceptable and consistent time span we restricted our data to the period from 2000 to 2019, except for three datasets relating to areas with unchanged land use. The first dataset was a global metanalysis dataset of soils under native land use (largely forestland)^5^ with a mean sampling year of 1992. As these samples were obtained from natural land uses, they were not expected to be influenced by anthropogenic phosphorus inputs. The second dataset involved 25 sites in Sahel and West African countries sampled in 1990^16^. Despite increases in green vegetation, land use intensification in these areas was very limited^17^. We therefore considered these soils to be representative of current practices. The third dataset included 17,920 values from the Second National Soil Farm Survey of China^18^ collected between 1980 and 1996. Major changes occurred in both the land use and land use intensity in eastern China, but not in western China, from 1986 to 2010^19–21^. To account for likely changes in the soil Olsen phosphorus concentration, we excluded data from the second survey prior to 1995 along with data pertaining to eastern provinces (Anhui, Fujian, Guangdong, Guangxi, Hainan, Hebei, Jiangsu, Jiangxi, Shandong, Sichuan, and Tianjin). We retained data originating from the remaining provinces where no land use change or intensification was noted^22–24^.

#### Step 3 Convert the data

We converted all data into soil Olsen phosphorus concentration data using regression equations suitable for Bray-I phosphorus, Resin-phosphorus, Kirsanov-phosphorus, AB-DPTA-phosphorus and for calcareous and non-calcareous soils^25^ considering Mehlich-3 phosphorus (Table 2). The slopes and coefficients of these equations were weighted according to the number of data points in each dataset. We note that pH can strongly affect these conversions especially if soil tests are used inappropriately; for example, using the Bray-I phosphorus test will dissolve calcium phosphates that are sparingly available to plants. We excluded Bray-I P data from soils ≥ pH 7 from our database. Moreover, while pH was found to have a minimal effect on conversions of Mehlich-3 phosphorus to Olsen phosphorus^26^, nevertheless we used separate equations for calcareous and non-calcareous soils (Table 2). The proportions of the total sites converted from Bray-I, Resin, Kirsanov, AB-DPTA and Mehlich-3 phosphorus at this stage were 50.7%, 0.4%, 0.6%, 0.1% and 4.8%, respectively, but after step 4, the proportions changed to 37.4%, 0.5%, 0.4%, 0.2%, and 4.7%, respectively. Nearly 57% of all the samples required no conversion (please refer to the filtering and conversion tabs in Final_Filtered_Raw_OlsenP_Plus_Predictors.xls or Steps_1_to_4.csv^27^).

#### Step 4 Adjustment to a consistent sampling depth and removal of duplicate values

Sites sampled at multiple depths were averaged to the top 20 cm, considering the proportion of a given sample within the top 20 cm and any variance in the bulk density at a certain depth^25^ (n = 11,756). For instance, if a sample was collected at depths from 15–25 cm, the sample influenced the mean value only within the 0–20 cm depth interval by a quarter (assuming all the soil samples exhibited the same bulk density). We did not make any adjustment for stratification of Olsen phosphorus concentrations in the deeper soil sample. However, much less stratification of Olsen phosphorus occurs with depth owing to strong sorption of phosphorus by the topsoil^28^. Where there were multiple concentrations for the same coordinates, we adopted the mean value (n = 176). Deeper samples and any duplicate values at a specific site and date were removed (n = 15,791).

**Fig. 2:**
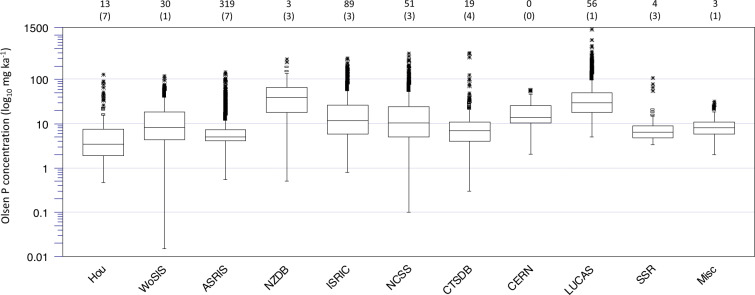
Box plots showing the 25^th^, 50^th^ and 75^th^ percentiles (top, middle and bottom of each box), the upper and lower fences (the 75^th^ and 25^th^ percentiles plus and minus 1.5 times the interquartile range, respectively) and minor (>75^th^ percentile but <upper fence) and major (>upper fence) Olsen P concentrations for each database. The values at the top indicate the number (and percentage in parentheses) of major outliers in each database.

Our final global dataset contained 33,102 values distributed across 89 countries, with a mean concentration of 26 mg kg^−1^. Over our sampling period, the mean sampling year was 2009 (Table 1). The percentage of major outliers (calculated as 1.5 times the interquartile range plus the upper fence of each database) varied from zero to seven (Fig. 2). However, when examining the whole database, the percentage of major outliers was <1%. We therefore did not remove outliers from the final database.

### Modelling

The filtered data (n = 32,941) were paired with predictor variables obtained from a wide variety of sources (Table 3). These predictor variables were chosen due to their high likelihood of influencing soil Olsen phosphorus stocks and included catchment characteristics, hydrological and climate parameters, land use, population, and ecological classifications^6,29^. We extracted data for each predictor variable from the sources outlined in Tables 3, 4 at a resolution of 1 km^2^, resulting in 933,120,000 points per variable considering the global land mass.

**Table 3:** Climatic variables and the units, years and sources of the variables used to predict the Olsen phosphorus concentration.

Variable	Unit	Year measured	Source
Mean monthly precipitation	mm	2010	^66^
Mean monthly temperature	°C	2010	^66^
Mean monthly maximum temperature	°C	2010	^66^
Mean monthly minimum temperature	°C	2010	^66^
Bioclimatic index 1 = Annual mean temperature	°C	2009	^66^
Bioclimatic index 2 = Mean diurnal range (mean monthly temperature (maximum temperature - minimum temperature))	°C	2009	^66^
Bioclimatic index 3 = Isothermal conditions (BIO2/BIO7) ( × 100)	—	2009	^66^
Bioclimatic index 4 = Temperature seasonality (standard deviation × 100)	—	2009	^66^
Bioclimatic index 5 = Maximum temperature of the warmest month	°C	2009	^66^
Bioclimatic index 6 = Minimum temperature of the coldest month	°C	2009	^66^
Bioclimatic index 7 = Annual temperature range (BIO5-BIO6)	°C	2009	^66^
Bioclimatic index 8 = Mean temperature of the wettest quarter	°C	2009	^66^
Bioclimatic index 9 = Mean temperature of the driest quarter	°C	2009	^66^
Bioclimatic index 10 = Mean temperature of the warmest quarter	°C	2009	^66^
Bioclimatic index 11 = Mean temperature of the coldest quarter	°C	2009	^66^
Bioclimatic index 12 = Annual precipitation	mm	2009	^66^
Bioclimatic index 13 = Precipitation of the wettest month	mm	2009	^66^
Bioclimatic index 14 = Precipitation of the driest month	mm	2009	^66^
Bioclimatic index 15 = Precipitation seasonality (coefficient of variation)	—	2009	^66^
Bioclimatic index 16 = Precipitation of the wettest quarter	mm	2009	^66^
Bioclimatic index 17 = Precipitation of the driest quarter	mm	2009	^66^
Bioclimatic index 18 = Precipitation of the warmest quarter	mm	2009	^66^
Bioclimatic index 19 = Precipitation of the coldest quarter	mm	2009	^66^

**Table 4:** Biophysical and geographic variables and the units, years and sources of the variables used to predict the Olsen phosphorus concentration.

Variable	Unit	Year measured	Source
Catchment area	km^2^		^67^
Enhanced vegetation index (EVI, monthly)	EVI at a 1-km^2^ resolution	2010	^68^
Normalised difference vegetation index (NDVI, monthly)	NVDI at a 1-km^2^ resolution	2010	^68^
Mean annual runoff	mm	2014	^69^
Slope	%		^67^
Altitude	m above sea level		^67^
Development status	Yes/no		^70^
Population density	Persons/km^2^	2000	^71^
Subsoil group number retrieved from SoilGrids	—	2016	^25^
Percentage of cropland	%	2000	^24^
Percentage of bare land	%	2015	^23^
Percentage of grassland or shrubland if not forestland, cropland or bare land	%	2015	^22–24^
Biome	—	2016	^72^
Country	—	2016	^70^

Prior to statistical analysis, log-transformed Olsen phosphorus concentrations were confirmed as approximately normally distributed with the Shapiro-Wilk test. A range of models was trialled to predict Olsen phosphorus concentrations. However, to minimise the likelihood that models were being overfitted we conducted a principal components analysis on 17 variables that were likely autocorrelated, being produced on a monthly timestep (e.g., EVI, NDVI, precipitation, mean temperature, mean maximum temperature, and mean minimum temperature). These components explained 96.9% of the variance in the set of variables and were all highly significant (*P* < 0.001) in the first model tried (a simple linear model) and so were included in all our models. Following the simple linear model, we developed a mixed effects model, then a random forest model, followed by generalised additive model (GAM) fitted with the mgcv^30^ procedure in R. Although the random forest model developed explained most of the most variance in the data, the computational requirements were too high for it to be applied on a global scale. We chose the implement the generalised additive model to predict log Olsen phosphorus concentrations, globally (Table 5).

**Table 5:** Approaches and performance metrics (Akaike Information Criterion, AIC; Nash Sutcliffe Efficiency, NSE) for each of the models tested.

Model approach	R squared (%)	AIC	NSE
Simple linear model	51	77345	0.204
Mixed-effect model	48	78317	0.214
Generalised additive model	54	75458	0.172
Random forest	68	-^1^	0.051

^1^AIC is not available for the Random Forest model as it has no log likelihood.

During modelling we used 70% of the data to train the models, while the remaining 30% was reserved to evaluate model performance. However, after finding little difference in predictive power between models using 70% or all the data, we chose to create the final model based on all the data.

It was not possible to predict values for the countries not included in the training data (representing 27.8% of global area). However, through the modelling process, country (geopolitical boundary) was an important predictor (see: R_model_outputs.docx^27^). To predict Olsen P concentrations for countries with no data, we randomly sampled 5% of each country in the dataset and renamed the country for those observations as “other” before rerunning the model. Thus, the “other” countries represented a weighted average of the countries present in the training data. This procedure may have biased the predictions for the “other” countries, as the model would be weighted towards countries with more training data, which may not be representative of those countries not represented in the training data. Users should be aware of this modelling fix and are advised to consult Country_counts.csv to judge the number of data points for each country.

Once models were run, predicted concentrations were back-transformed and corrected for the retransformation bias with the smearing estimate method^31^:

$$S=\frac{1}{n}{\sum }_{i=1}^{n}{e}^{\widehat{{\varepsilon }_{i}}}$$

where *ε*_*i*_ denotes the residuals of the regression models. The correction factor (*S*) is applied over the whole range of predictions, as it is assumed that the residuals are homoscedastic.

The back-transformed predictions of Olsen phosphorus concentrations in topsoil were projected globally in ArcGIS. Raster grids were created at a spatial resolution of 0.025 degrees (ca. 1 km^2^ near the equator), which corresponds to the coarsest grid cells associated with the input data, as listed in Tables 3 and 4.

### Post-processing adjustments

Our preliminary modelling established that the biome and development status of a given country were important factors influencing the projection of Olsen phosphorus concentration in that country (see: R_model_outputs.docx^27^). However, most of the data used to generate our global model were derived from developed regions and productive biomes. To determine if large areas were being modelled with a paucity of data, we split the database into biomes and whether a data point pertained to a developing or underdeveloped country. After inspecting the data, we found that five biomes suffered from a paucity of data (n < 100): deserts (within the desert and xeric shrubland biome); flooded grasslands and savannas and mangroves (developed); tropical and subtropical dry broadleaf forests, and montane grasslands and shrublands (underdeveloped). These biomes represented 12.6%, 0.9%, <0.1%, 3.7% and 0.5%, respectively, of the global land area, but are all largely unproductive. As previous studies have identified lower but stable soil phosphorus concentrations in unproductive biomes than in productive biomes, we used literature data to replace our modelled estimates of soil Olsen phosphorus. These biomes were assigned Olsen phosphorus concentrations (mg kg^−1^) of 2.0^32^, 5.4^33^, 3.5^34^, 3.1^35^ and concentrations between 1 and 3 depending on slope and elevation^36^ (see: Final_Filtered_Raw_OlsenP_Plus_Predictors.xlsx; post modelling processing tab or Steps_1_to_4.csv^27^).

Few data were available for South Africa. However, a prior spatial model of the mean soil available phosphorus (Bray-I phosphorus) in South Africa was available at the provincial level^37^. This model was generated from >10,000 data points and performed better (for South Africa) than our model (r^2^ = 0.68 *cf*. 0.54). Hence, we converted the modelled South African Bray-I phosphorus concentrations at a provincial level into Olsen phosphorus concentrations and applied it instead of ours.

### Calculation and soil Olsen phosphorus stocks

**Fig. 3:**
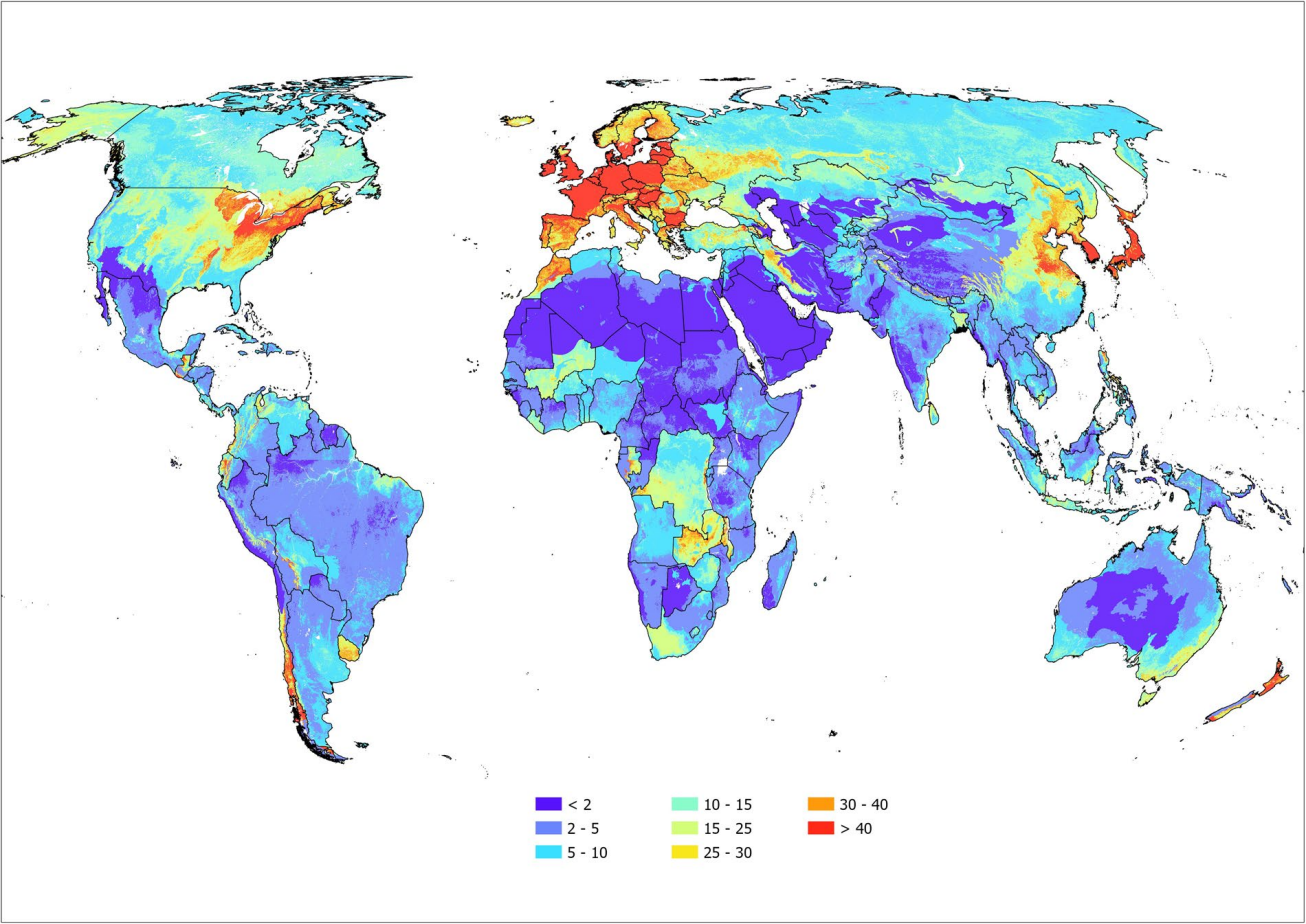
Global topsoil Olsen phosphorus concentration (mg kg^−1^). The mapped land parcels are plotted at a resolution of 1-km^2^ and were calculated from a database containing *ca*. 575,000 soil samples of freely available data with a wide geographic coverage. An interactive version of this map, allowing users to discover predicted concentrations at selected points is available at: https://world-olsen.agr.nz/.

To predict soil Olsen phosphorus stocks, the predicted concentration data (Fig. 3) were multiplied by bulk density data^25^. Predicted Olsen concentration and bulk density data were assumed to cover 1-km^2^ land parcels with a topsoil thickness of 20 cm. The mass in each pixel was calculated in kilotons. The predicted global stock (across 136 M km^2^ of land) is estimated to be 318,618 kt (±21,985 kt), while continental stocks are estimated to be: 47,847 (±3,301), 86.474 (±4,483), 84,401 (±7,279), 60517 (±4,176), 13,374 (±951), and 26,005 kt (±1,795 kt), for Africa, Asia, Europe, North America, Oceania, and South America, respectively. Variation in stocks were calculated as the coefficient of variation using ${\widehat{cv}}_{raw}=\sqrt{{e}^{{s}_{ln}^{2}}-1}$ for each estimate in the dataset^38^ and the “metrumrg” package in R^39^ (see also R_code_output.docx). The mean coefficient of variation was 0.069 or 6.9%. The stocks and area calculated for each continent (and country) are given in Stats_by_Continent.xlsx. The mean stock for countries was 1356 kt, ranging from <1 for small Caribbean Island nations to 39267 kt for the US.

**Fig. 4:**
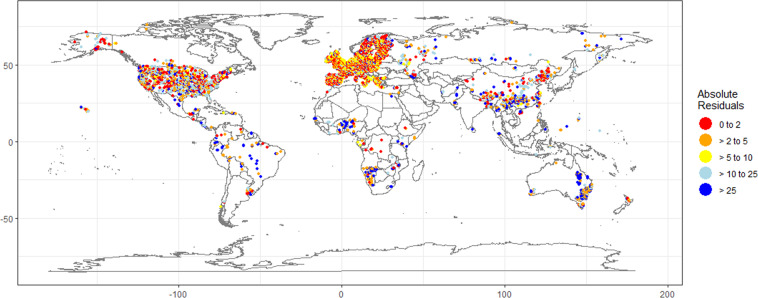
Map of the residuals for each data point calculated as the difference between GAM predictions and the original value and classed the percentage difference into five classes: 0–2, 2.1–5, 5.1–10, 10.1–25 and >25%.

On average the percentage difference between the predicted and observed data (i.e., residuals) was 14.9%. We classed the percentage differences into 0–2, 2.1–5, 5.1–10, 10.1–25 and >25%. The percentage of our predictions that were in each class was 14, 19, 20, 30 and 18%, respectively (see Final_Filtered_Raw_OlsenP_Plus_Predictors.xlsx Residuals by continent tab or Residuals_by_continent.csv^27^). A map of the percentage differences is given in Fig. 4.

## Data Records

The data and code used in modelling and outputs are available in Figshare^27^. A list of the data files and outputs is available in Supplementary Table 1.

## Technical Validation

### Validating conversions to Olsen phosphorus

**Fig. 5:**
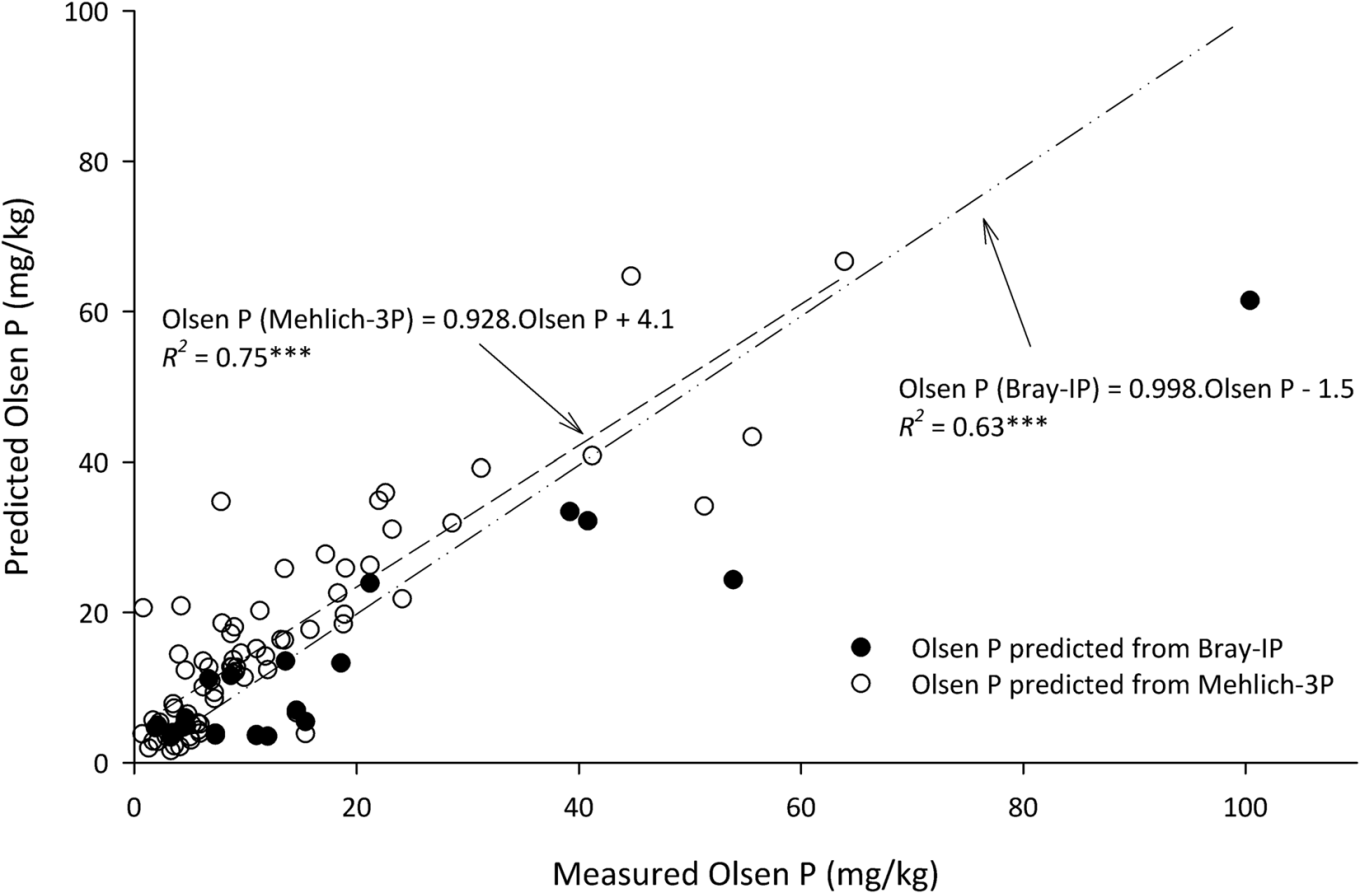
Validation of Olsen phosphorus (P) predictions via the equations for Bray-IP and Mehlich-3P in Table 2 and independently sourced data from the NCSS. In addition to a significant fit (*P* < 0.001), and slope approaching 1, the Nash Sutcliffe Efficiency was >0.7 for each regression.

The conversions from Mehlich-3 P or Bray-I P to Olsen phosphorus were validated against the National Cooperative Soil Survey database, which contains observations of both Olsen phosphorus and Mehlich-3 P or Bray-I P for 97 samples. With the use of equations for either Bray-I P (Olsen phosphorus = 0.49 × Bray-I P + 3.1) or Mehlich-3 P (Olsen phosphorus = 0.47 × Mehlich-3 P + 2.4 for non-calcareous soils and Olsen phosphorus = 0.41 × Mehlich-3 P + 1.1 for calcareous soils), we predicted Olsen phosphorus concentrations and compared these estimates to measured Olsen phosphorus concentrations. The regression outputs (*P* < 0.001) indicate that the slope between the measured and predicted values approaches 1 (0.998 for Bray-I P and 0.928 for Mehlich-3 P; Fig. 5), suggesting that the equations are suitable for general use as a conversion tool.

### Validating soil Olsen phosphorus stocks

We compared our estimate of the topsoil Olsen phosphorus stock in sub-Saharan Africa to the previously modelled and published phosphorus stock in Sub-Saharan Africa. These published stocks were expressed as Mehlich-3 phosphorus^6^, so we converted Mehlich-3 phosphorus stocks to Olsen phosphorus stocks for 1-km^2^ parcels of calcareous and non-calcareous soils using the equations provided in Table 2. Excluding the Saharan Desert^23^, our modelled estimate was 36,875 kt of Olsen phosphorus for the 0–20 cm depth. After converting the published stock of Mehlich-3 phosphorus (estimated for the 0–30 cm depth) into Olsen phosphorus by the equations in Table 2, the Olsen phosphorus stock was 28,890 kt; our estimate was 27% greater, but 1% greater if the stock for the forested land was also removed.

## Usage Notes

These data and the estimated global distribution of soil Olsen phosphorus stocks can be used to estimate where soil Olsen phosphorus is deficient or more than required for optimal crop growth. This can guide more efficient use of fertiliser stocks and can also indicate the potential for phosphorus loss from land to water, for example via erosion, which can impair water quality through eutrophication^12^. However, it should be noted that such assessments are best done at a continental scale or at most a country or basin scale owing to the paucity of data in some regions, leading to high variability in the modelled stocks. It is advised that work requiring soil Olsen phosphorus stocks for policy at smaller scales therefore be supported by more localised sampling.

## Supplementary information

Supplementary Table 1

## Data Availability

The following code and outputs are available on-line^27^: • The R code and outputs of the efficacy and performance of the models employed to estimate the global Olsen phosphorus concentration from the predictor variables. • Python code describing the filtering and post-processing steps involved in the use and analysis of the raw data and predictor variables to estimate global soil Olsen phosphorus concentration values and stocks.
