# Supplementary material for: A Global Database of Soil Plant Available Phosphorus
Source: Sci Data. 2023 Mar 7;10:125. doi: 10.1038/s41597-023-02022-4 (PMC9992394; doi:10.1038/s41597-023-02022-4)
Supplement: Supplementary file 1 — Supplementary Table 1 [file 41597_2023_2022_MOESM1_ESM.docx]

**Supplementary Table 1**. List of the input data files, predictors, code and outputs used to generate the global map of soil Olsen phosphorus concentrations.

| File ID | Description |
| --- | --- |
| ASRIS_clean.csv | Data cleaned and filtered from the Australian Soil Resources Information System |
| CERN_clean.csv | Data cleaned and filtered from the Chinese Ecosystem Research Network |
| CERN_raw.csv | Unfiltered data from the |
| Country_counts.csv | Count of data from each country in the final cleaned and filtered database |
| Country_stocks_by_continent.csv | Stocks of Olsen P for each country and continent |
| CTSDB_clean.csv | Data cleaned and filtered from the Soil database for land surface modelling |
| CTSDB_raw.csv | Unfiltered data from the |
| Final_filtered_data.csv | Final cleaned and filtered Olsen P database plus predictors for modelling |
| Final_Filtered_Raw_OlsenP_Plus_Predictors.xlsx | Presents the raw data, the filtering and evaluation procedures outlined in steps 1 to 4 and the aligned predictor variables for estimating global Olsen phosphorus concentrations. Metadata are provided for all tabs |
| Hou_clean.csv | Data cleaned and filtered from the global dataset of plant-available P |
| Hou_raw.csv | Unfiltered data from the global dataset of plant-available P |
| ISRIC_clean.csv | Data cleaned and filtered from the International Soil Reference and Information Centre |
| ISRIC_raw.csv | Unfiltered data from the International Soil Reference and Information Centre |
| LUCAS_clean.csv | Data cleaned and filtered from the Land Use/Land Cover Area Frame Survey Topsoil Survey |
| LUCAS_raw.csv | Unfiltered data from the Land Use/Land Cover Area Frame Survey Topsoil Survey |
| Metadata.csv | Metadata for the final cleaned and filtered database and all cleaned and filtered contributing databases |
| Misc.csv | Data cleaned and filtered from a range of publications |
| NSCC_clean.csv | Data cleaned and filtered from the National Cooperative Soil Survey |
| NSCC_raw.csv | Unfiltered data from the National Cooperative Soil Survey |
| NZDB_clean.csv | Data cleaned and filtered from the New Zealand Soil Database |
| OlsenP_kgha1_World_Aug2022.tif | Geo-referenced file of Olsen phosphorus stocks at 1-km^2^ resolution |
| Post_modelling_processing.csv |  |
| R_Code.Rmd | R code for each of the models tested to predict Olsen phosphorus concentrations |
| R_model_outputs.docx | Outputs of the performance of each model tested to predict Olsen phosphorus concentrations and the residuals for the Generalised Additive Model. |
| Residuals_by_continent.csv | Count and percentage of predicted values that were within classes of different percentage residuals |
| SSR.csv | Data cleaned and filtered from the Soil Resources of Russia |
| Steps_1_to_4.csv | Raw data removed during steps 1 to 4 of the cleaning and filtering process |
| Stocks_by_continent.xlsx | Present Olsen P stocks for each country and land use. Note that this file also contains a graphical representation of the data as a “3D Map”. |
| WoSIS_clean.csv | Data cleaned and filtered from the World Soil Information Service |
| WoSIS_raw.csv | Unfiltered data from the World Soil Information Service |
